# Supplementary material for: Zoology of Multiple‐Q Spin Textures in a Centrosymmetric Tetragonal Magnet with Itinerant Electrons
Source: Adv Sci (Weinh). 2022 Jan 28;9(10):2105452. doi: 10.1002/advs.202105452 (PMC8981443; doi:10.1002/advs.202105452)
Supplement: Supplementary file 1 — Supporting Information [file ADVS-9-2105452-s001.pdf]

ADVANCED SCIENCE
Open Access

Supporting Information

for *Adv. Sci.*, DOI 10.1002/advs.202105452

Zoology of Multiple-*Q* Spin Textures in a Centrosymmetric Tetragonal Magnet with Itinerant Electrons

Nguyen Duy Khanh, Taro Nakajima, Satoru Hayami, Shang Gao, Yuichi Yamasaki, Hajime Sagayama, Hironori Nakao, Rina Takagi, Yukitoshi Motome, Yoshinori Tokura, Taka-hisa Arima and Shinichiro Seki**

ADVANCED SCIENCE
Open Access

Supporting Information

for *Adv. Sci.,* DOI: 10.1002/advs.202105452

Zoology of multiple-*Q* spin textures in a centrosymmetric tetragonal magnet with itinerant electrons

Nguyen Duy Khanh, Taro Nakajima, Satoru Hayami, Shang Gao, Yuichi Yamasaki, Hajime Sagayama, Hironori Nakao, Rina Takagi, Yukitoshi Motome, Yoshinori Tokura, Taka-hisa Arima, Shinichiro Seki**

Supplementary Information: Zoology of multiple-Q spin textures in a centrosymmetric tetragonal magnet with itinerant electrons

Nguyen Duy Khanh[1,2,*], Taro Nakajima[1,2], Satoru Hayami[3], Shang Gao[1,10],
Yuichi Yamasaki[4,5], Hajime Sagayama[6], Hironori Nakao[6], Rina Takagi[3,5,8],
Yukitoshi Motome[3], Yoshinori Tokura[1,3,9], Taka-hisa Arima[1,7], Shinichiro Seki[1,3,5,8,*]

[1] *RIKEN Center for Emergent Matter Science (CEMS), Wako, Japan*

[2] *Institute for Solid State Physics (ISSP), University of Tokyo, Kashiwa, Japan*

[3] *Department of Applied Physics, The University of Tokyo, Tokyo, Japan*

[4] *Research and Services Division of Materials Data and Integrated System (MaDIS), National Institute for Materials Science (NIMS), Tsukuba, Japan*

[5] *PRESTO, Japan Science and Technology Agency (JST), Kawaguchi, Japan*

[6] *Institute of Materials Structure Science, High Energy Accelerator Research Organization, Tsukuba, Ibaraki, Japan*

[7] *Department of Advanced Materials Science, The University of Tokyo, Kashiwa, Japan*

[8] *Institute of Engineering Innovation, The University of Tokyo, Tokyo, Japan*

[9] *Tokyo College, The University of Tokyo, Tokyo, Japan and*

[10] *Current affiliation: Materials Science & Technology Division, Oak Ridge National Laboratory, Oak Ridge, TN, USA and Neutron Science Division, Oak Ridge National Laboratory, Oak Ridge, TN, USA**

1. H-T MAGNETIC PHASE DIAGRAM FOR $\mathbf{H} \parallel [100]$

In Supplementary Figs. S1b-d, magnetic field dependence of magnetization M, longitudinal resistivity ρ_{xx} and Hall resistivity ρ_{yx} measured at 5 K for $\mathbf{H} \parallel [100]$ are plotted. In these profiles, we can identify clear anomalies at 2 T, 4 T, 10 T, and 11 T, which represent the $\mathbf{H}$-induced metamagnetic transitions among the phases I $\rightarrow$ IV $\rightarrow$ III' $\rightarrow$ V $\rightarrow$ FM (i.e. the ferromagnetic state with saturated magnetization $M \sim 7\ \mu_B/Gd^{3+}$). By performing the similar measurements at various temperatures, H-T magnetic phase diagram for $\mathbf{H} \parallel [100]$ has been summarized as shown in Supplementary Figure S1a. Note that the obtained phase boundaries are almost consistent with the previous report[S2], while the appearance of the phase V at high field region has newly been identified in the present work. The observed non-monotonous ρ_{yx} profile in Supplementary Figure 1d probably reflects the complicated H-dependence of ρ_{xx}, as well as the change of electronic structure upon the metamagnetic transition. It may also contain the possible contribution of chiral Hall effect recently proposed in [S1]. Its quantitative analysis is the issue for the future study.

2. MAGNETIC PHASE DIAGRAM AS A FUNCTION OF $H_{[100]}$ AND $H_{[001]}$

In Supplementary Fig. S2a, M-H profiles measured at 5 K for various magnetic field directions are plotted. Here, the $\mathbf{H}$-direction is confined within the (010) plane, and θ represents the angle between the $\mathbf{H}$-direction and the [001] axis. At $\theta = 0^\circ$ ($\mathbf{H} \parallel [001]$), magnetization curve exhibits clear step-like anomalies at $\mu_0 H = 2$ T and 2.6 T, corresponding to the metamagnetic transitions among the phases I $\rightarrow$ II $\rightarrow$ III. On the other hand, at $\theta = 90^\circ$ ($\mathbf{H} \parallel [100]$), anomalies are observed at 2 T and 4 T corresponding to the transitions between the phases I $\rightarrow$ IV $\rightarrow$ III'. On the basis of those magnetization profiles, the magnetic phase diagram as a function of $H_{[100]}$ and $H_{[001]}$ is summarized in Supplementary Fig. S2b.

In Supplementary Figs. S2c-e, the θ-dependence of $\mathbf{M}$ measured at 7 T, 3 T and 1 T are plotted. The corresponding polar plots are also indicated in Supplementary Figs. S2f-h. At 3 T, a series of clear step-like anomalies are observed, which corresponds to the transitions between the phases III and IV. At 7 T, on the other hand, the magnetization profile shows smooth sinusoidal θ-dependence, suggesting the continuous transformation between the phase III and III' as a function of θ.

3. RESONANT X-RAY SCATTERING PROFILES FOR H ∥ [100]

In the following, we discuss the development of magnetic modulation vector **Q** based on the RXS profiles measured at various amplitudes of **H** ∥ [100]. Supplementary Figs. S3a and S3b indicate the line scan profiles along the (δ, 4, 0) and (0, 4-τ, 0) directions, which allows the identification of the magnetic modulation vectors $\mathbf{Q}_1 = (q, 0, 0)$ and $\mathbf{Q}_2 = (0, q, 0)$ that are parallel and perpendicular to the external magnetic field **H**, respectively (These figures correspond to detailed version of Figs. 2a and 2b in the main text, with enhanced number of magnetic field data points). Here, the sample was initially cooled at $\mu_0 H = 0$ T, and the data points represented by open symbols were first obtained in the field increasing process from 0 T to 5 T. Then, the ones represented by closed symbols were obtained in the field decreasing process from 5 T to 0 T. After the initial zero-field-cooling (i.e. the phase I), the magnetic

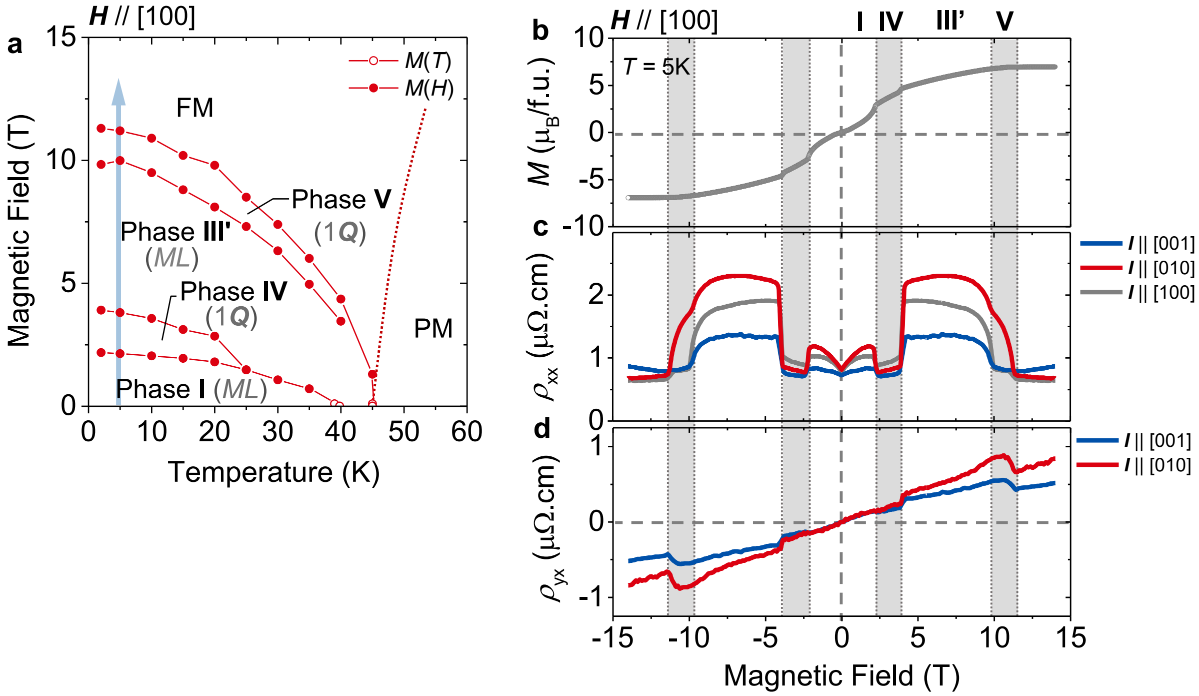

FIG. S1. H-T magnetic phase diagram for **H** ∥ [100]. a) H-T magnetic phase diagram of $GdRu_2Si_2$ for **H** ∥ [100] based on the anomalies in temperature (T) and magnetic field (H) dependence of magnetization M. b-d) Magnetic field dependence of magnetization M, longitudinal resistivity ρ_{xx} and Hall resistivity ρ_{yx} measured at 5 K for **H** ∥ [100]. For the measurement of ρ_{xx} and ρ_{yx}, electric current I is applied along various directions, and each data is plotted by different colors.

satellite reflections are found for both [100] and [010] directions. By applying $\mathbf{H} \parallel [100]$, the phase IV is stabilized for $2\ \mathrm{T} < \mu_0 H < 4\ \mathrm{T}$, where the magnetic satellite reflection is observed only along the direction parallel to $\mathbf{H}$. This result indicates that the phase IV is the single-$\mathbf{Q}$ state with $\mathbf{Q}_1 \parallel \mathbf{H}$. Above 4 T (i.e. phase III'), the magnetic satellite reflections reappear along the both directions parallel and perpendicular to $\mathbf{H}$, which suggests that the phase III' is a double-$\mathbf{Q}$ state. When the H-value is reduced from this situation, only $\mathbf{Q}_1 \parallel \mathbf{H}$ survives in the phase IV, but then both $\mathbf{Q}_1 \parallel \mathbf{H}$ and $\mathbf{Q}_2 \perp \mathbf{H}$ reappear in phase I. Here, the phase I is characterized by the anisotropic pattern of magnetic satellite reflections with $\mathbf{Q}_1 = (0.219, 0, 0)$ and $\mathbf{Q}_2 = (0, 0.224, 0)$. This indicates that the phase I is a double-$\mathbf{Q}$ state with anisotropic character, and the application of $\mathbf{H} \parallel [100]$ selects one of equivalent magnetic domains associated with the four-fold symmetry breaking.

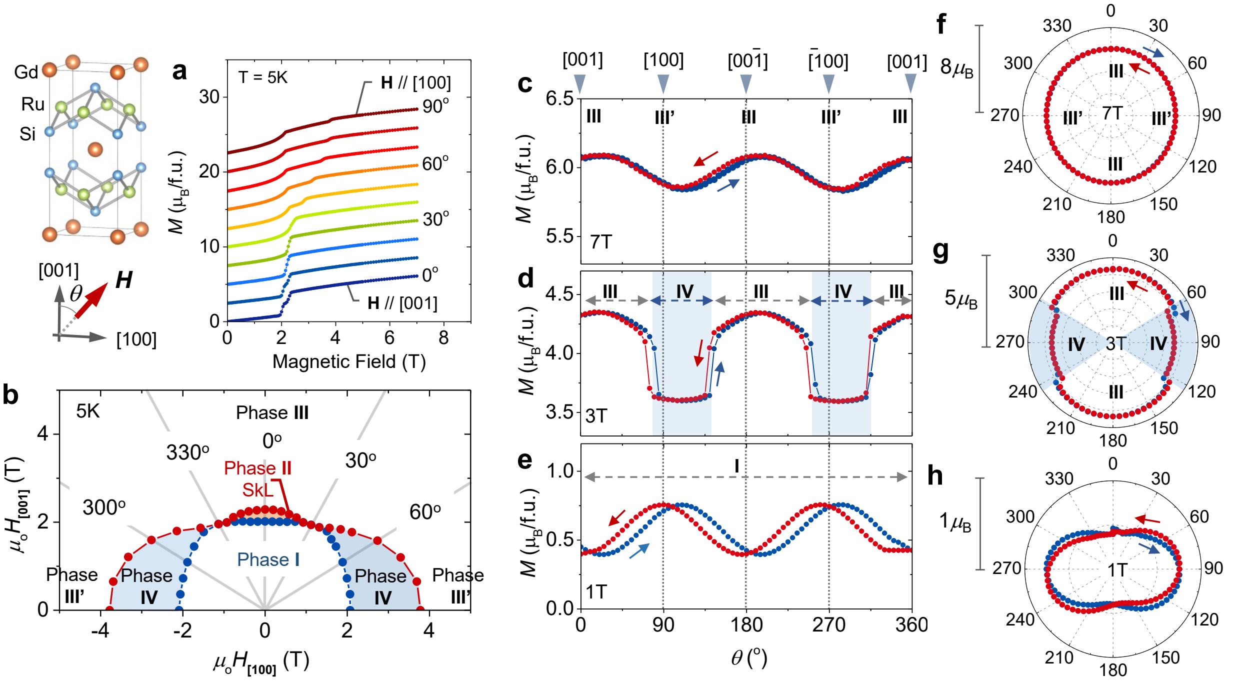

FIG. S2. Magnetic phase diagram as a function of $H_{[100]}$ and $H_{[001]}$. a) Crystal structure of $GdRu_2Si_2$ and M-H profiles measured at 5 K for various magnetic field direction. Here, the $\mathbf{H}$-direction is confined within the (010) plane, and θ represents the angle between the $\mathbf{H}$-direction and the [001] axis. b) Magnetic phase diagram as a function of $H_{[100]}$ and $H_{[001]}$ at 5 K, summarized based on the magnetization profiles in (a). c-e) θ-dependence of $\mathbf{M}$, measured at $\mu_0 H = 7$ T, 3 T and 1 T. Corresponding polar plots are shown in (f-h).

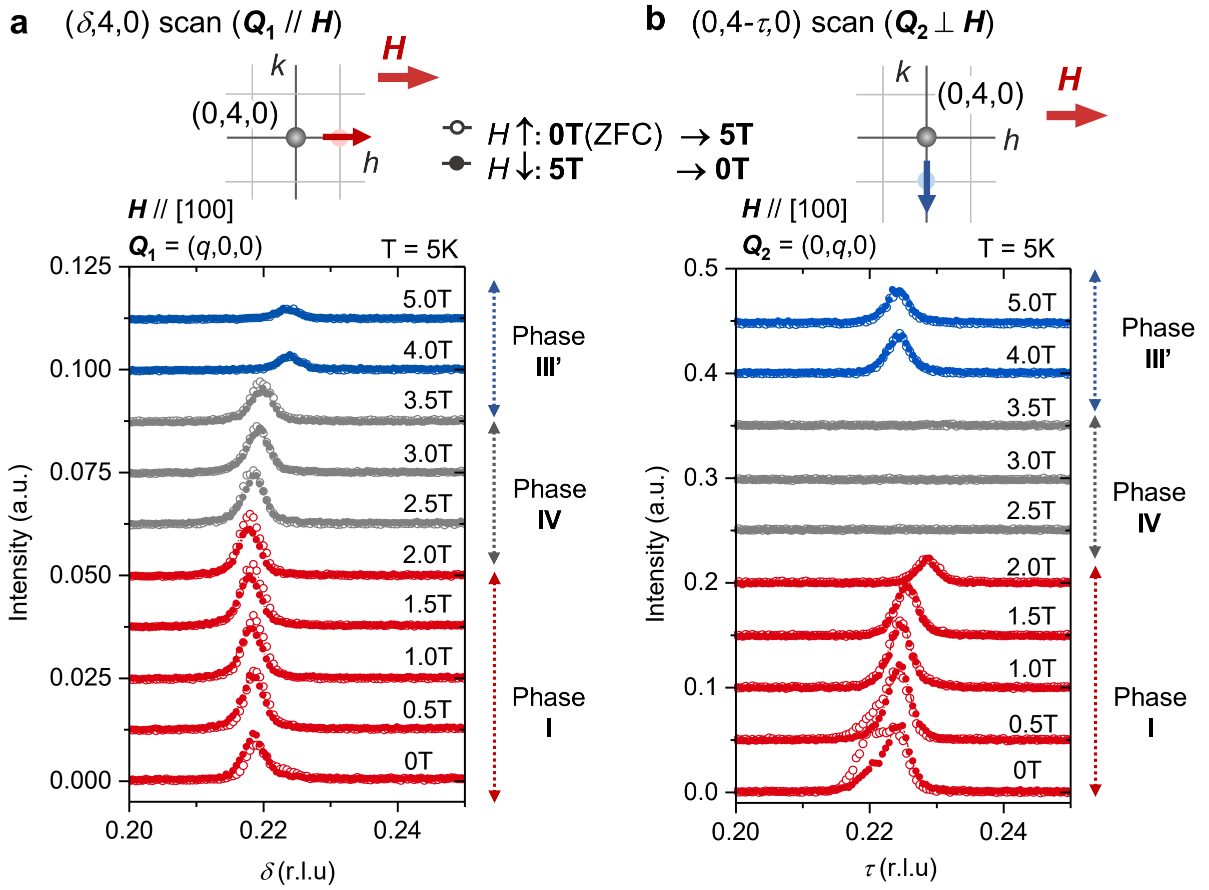

FIG. S3. Magnetic field dependence of RXS profiles of $\mathbf{Q_1}$ and $\mathbf{Q_2}$ magnetic satellite peaks for $\mathbf{H} \parallel [100]$. a) Line profiles for ($\delta$, 4, 0) scan measured with various amplitude of $\mathbf{H} \parallel [100]$ at 5 K, which represents the $\mathbf{Q}_1$ ($\parallel \mathbf{H}$) magnetic satellite peak around the fundamental Bragg spot (0, 4, 0). The sample was initially cooled at $\mu_0 H = 0$ T, and the data points represented by open symbols are first measured in the field increasing process from 0 T to 5 T. Then, the one represented by closed symbols are measured in the field decreasing process from 5 T to 0 T. The schematic illustration of line-scan direction in the reciprocal space is shown in the upper panel. b) The corresponding data for (0, $4-\tau$, 0) scan, representing the $\mathbf{Q}_2$ ($\perp \mathbf{H}$) magnetic satellite peak.

4. MAGNETIC STRUCTURE ANALYSIS IN THE PHASE IV FOR H ∥ [100]

In this part, we investigate the spin texture in the phase IV ($\mu_0 H = 3$ T, $T = 5$ K), which represents a single-$\mathbf{Q}$ state with $\mathbf{Q}_1 \parallel \mathbf{H} \parallel [100]$ as confirmed in the Supplementary Note III. For this purpose, the polarization analysis of scattered X-ray has been performed for the magnetic satellite reflections at $(0,4,4) \pm \mathbf{Q}_1$, with the experimental geometry shown in Supplementary Figs. S4a and S4b (which is the same as the one employed in Figs. 3b

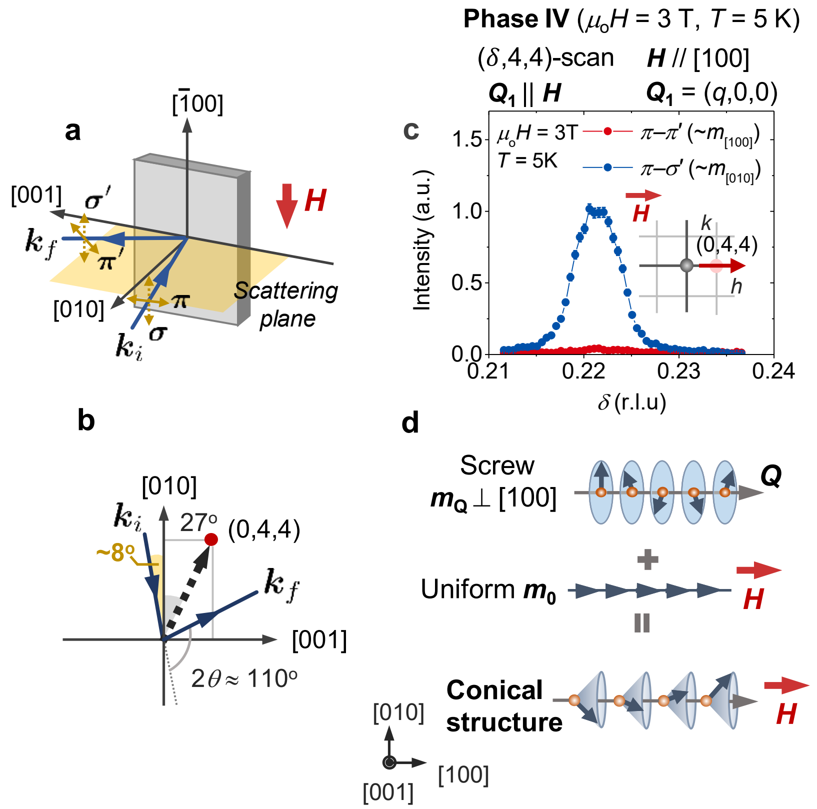

FIG. S4. Polarization analysis of RXS profile in the phase IV for $\mathbf{H} \parallel [100]$. a,b) Experimental configuration of RXS measurement to investigate magnetic satellite peaks around the fundamental Bragg spot (0, 4, 4), which is the same as the one employed in Figs. 3b and c in the main text. Scattering plane is always perpendicular to the [100] axis. $\mathbf{k}_i$ and $\mathbf{k}_f$ are the propagation vectors of incident and scattered X-ray. $\mathbf{k}_i$ is almost parallel to the $[0\bar{1}0]$ direction. c) Line profiles for (δ, 4, 4) scans corresponding to $\mathbf{Q}_1$ ($\parallel \mathbf{H}$), measured at 5 K with $\mu_0 H = 3$ T applied along the [100] direction (phase IV). d) Schematic illustration of the conical magnetic structure.

and 3c in the main text). Here, the incident X-ray direction $\mathbf{k}_i$ is almost parallel to the $[0\bar{1}0]$ direction, and $I_{\pi-\pi'}$ and $I_{\pi-\sigma'}$ mainly reflect the [100] and [010] component of $\mathbf{m}_{\mathbf{Q}_1}$, respectively, according to Eq. (2) in the main text. Supplementary Fig. S4c indicates the line scan profile of $I_{\pi-\pi'}$ and $I_{\pi-\sigma'}$ for $\mathbf{Q}_1$. The observed existence of $I_{\pi-\sigma'}$ and absence of $I_{\pi-\pi'}$ suggest that $\mathbf{m}_{\mathbf{Q}_1}$ contains the [010] component, but not the [100] component. On the basis of the above results and by considering the weak easy-axis magnetic anisotropy[S2] and fixed amplitude of localized magnetic moment at Gd^{3+} site, we suppose that the phase IV should accompany the screw type modulation, where the neighboring spins rotate within a plane perpendicular to the magnetic modulation vector $\mathbf{Q}_1 \parallel \mathbf{H}$. Since the uniform ferromagnetic component along the [100] direction is further induced by $\mathbf{H}$, the resultant spin texture can be considered as the conical one, as shown in Supplementary Fig. S4d.

5. MAGNETIC STRUCTURE ANALYSIS IN THE PHASE V FOR H || [100]

In this part, we investigate the magnetic structure in the phase V induced by $\mathbf{H} \parallel [100]$. We performed the resonant X-ray scattering experiments at 35 K to access to the phase V (Supplementary Fig. S5c), since 7 T is the the upper limit of magnetic field in the present setup. First, we examined the development of magnetic modulation vector $\mathbf{Q}$ in $\mathbf{H} \parallel [100]$, by exploring the magnetic satellite reflections around the fundamental Bragg peak indexed as $(0, 4, 0) \pm \mathbf{Q}$. Supplementary Figs. S5a and S5b indicate the line scan profiles along the (δ, 4, 0) and (0, 4-τ, 0) directions, which allows the identification of the magnetic modulation vectors $\mathbf{Q}_1 = (q, 0, 0)$ and $\mathbf{Q}_2 = (0, q, 0)$ that are parallel and perpendicular to the external magnetic field $\mathbf{H}$, respectively. The corresponding H-dependence of integrated intensities for $\mathbf{Q}_1$ and $\mathbf{Q}_2$ magnetic satellite reflection are also plotted in Supplementary Fig. S5d. After the initial zero-field-cooling (i.e. the phase I), the magnetic satellite reflections are found for both [100] and [010] directions. By applying $\mathbf{H} \parallel [100]$, successive metamagnetic transitions in order of I $\rightarrow$ III' $\rightarrow$ V $\rightarrow$ FM are induced. In the middle panel of Supplementary Figs. S5a and b, the scattering profiles in the phase V measured at 5.4 T are indicated, where the magnetic satellite reflection is observed only along the direction perpendicular to $\mathbf{H}$. This result indicates that the phase V is a single-$\mathbf{Q}$ state with $\mathbf{Q}_2 \perp \mathbf{H}$.

To investigate the detailed spin texture in the Phase V, we have performed the polarization analysis of the scattered X-ray in the RXS experiments. First, the magnetic satellite

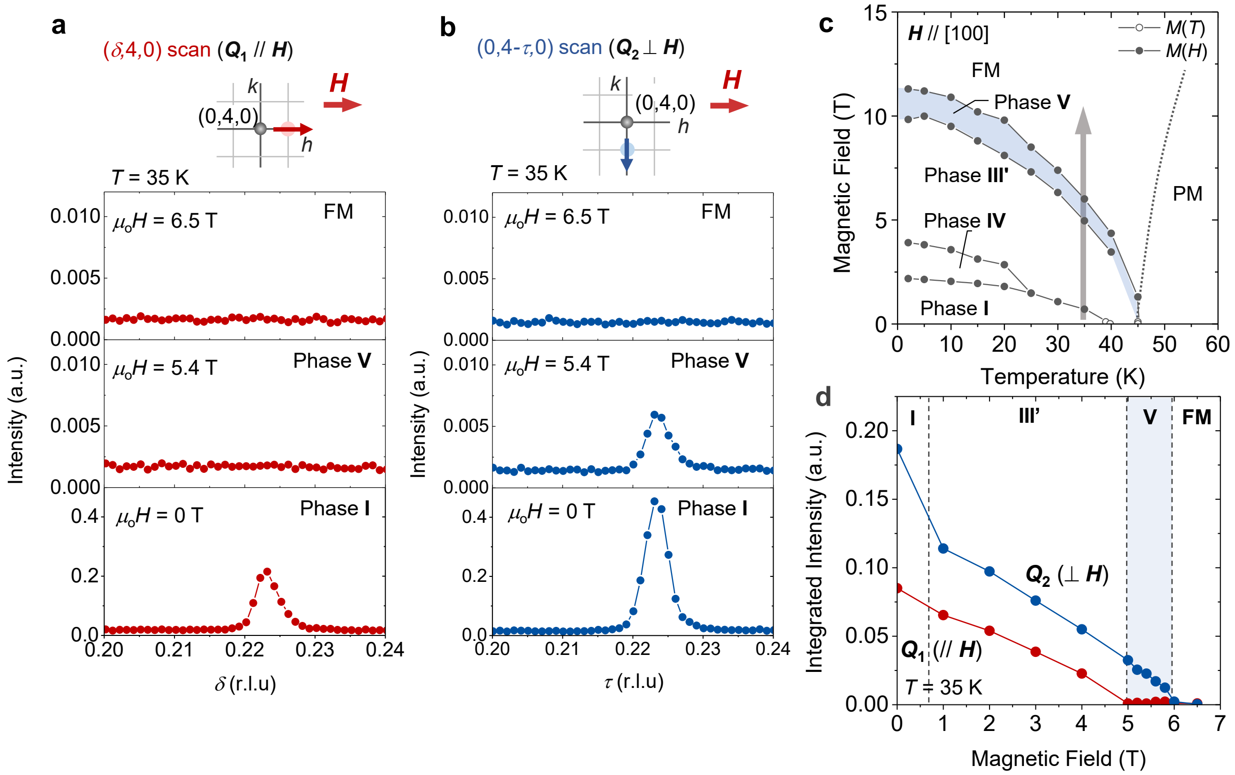

FIG. S5. Resonant X-ray scattering profiles in the phase V under $\mathbf{H} \parallel [100]$. a) Line profiles for ($\delta$, 4, 0) scan measured with various amplitude of $\mathbf{H} \parallel [100]$ at 35 K, which represents the $\mathbf{Q}_1$ ($\parallel \mathbf{H}$) magnetic satellite peak around the fundamental Bragg spot (0, 4, 0). All the data are measured in the field-increasing process after the zero field cooling. The schematic illustration of line-scan direction in the reciprocal space is shown in the upper panel. b) The corresponding data for (0, $4-\tau$, 0) scan, representing the $\mathbf{Q}_2$ ($\perp \mathbf{H}$) magnetic satellite peak. c) Magnetic phase diagram for $\mathbf{H} \parallel [100]$. The arrow indicates the path of magnetic field scan. d) Magnetic field dependence of integrated intensity for $\mathbf{Q}_1$ ($\parallel \mathbf{H}$) and $\mathbf{Q}_2$ ($\perp \mathbf{H}$) magnetic satellite reflections.

peaks at $(0, 4, 0) \pm \mathbf{Q}_2$ are investigated with the setup shown in Supplementary Fig. S6a. In this configuration, the propagation vector of incident x-ray $\mathbf{k}_i$ is almost parallel to the $[0\bar{1}1]$ direction, and Eq. (2) in the main text suggests that $I_{\pi-\pi'}$ and $I_{\pi-\sigma'}$ mainly reflect the [100] and $[0\bar{1}1]$ projection component of $\mathbf{m}_{\mathbf{Q}_2}$, respectively. Supplementary Fig. S6c indicates the line scan profiles of $I_{\pi-\pi'}$ and $I_{\pi-\sigma'}$ intensities for $\mathbf{Q}_2 = (0,\, q,\, 0)$. The observed presence of $I_{\pi-\sigma'}$ and absence of $I_{\pi-\pi'}$ suggest that $\mathbf{m}_{\mathbf{Q}_2}$ contains [010] or [001] component, but not the [100] component. We have further performed the similar polarization analysis for the

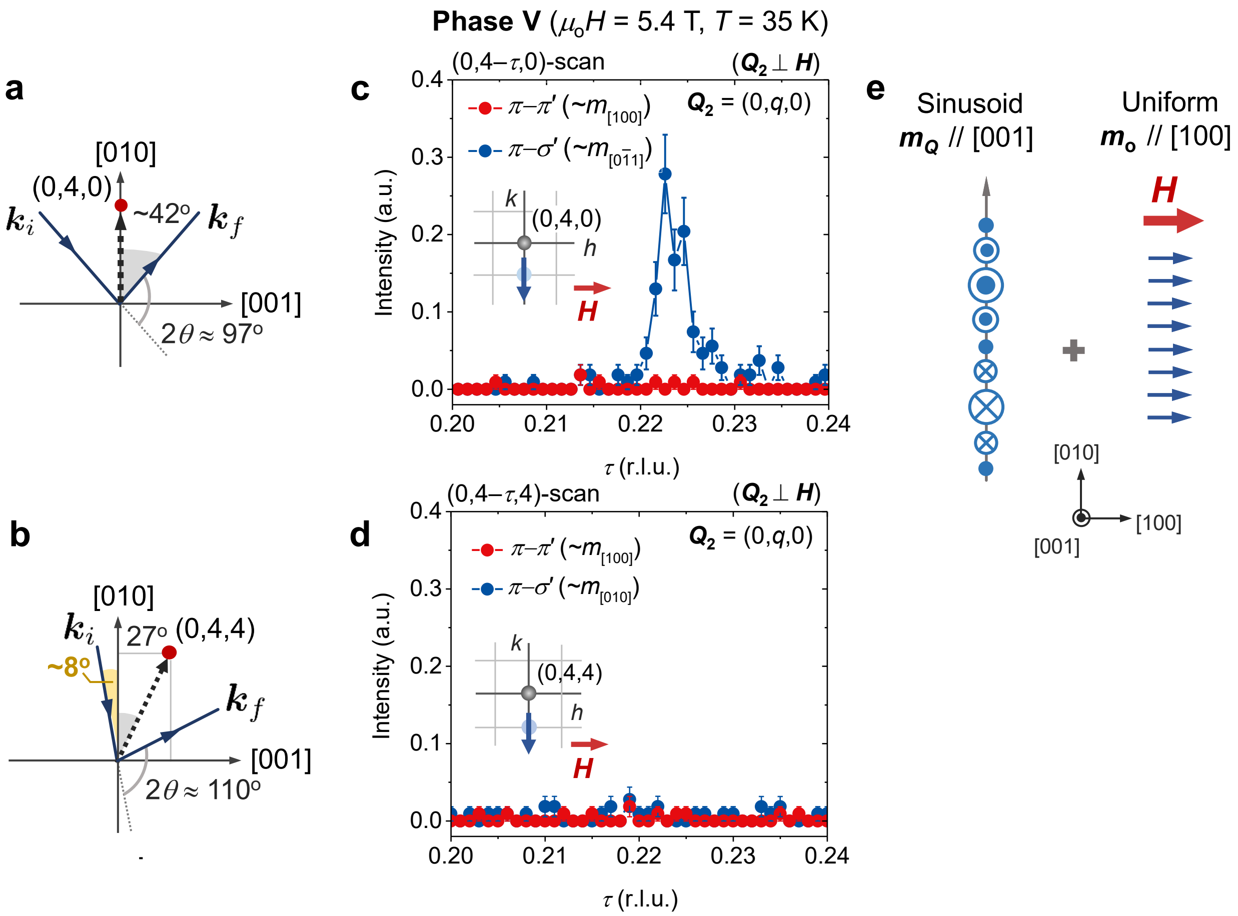

FIG. S6. Polarization analysis of RXS profile in the phase V for $\mathbf{H} \parallel [100]$. a,b) Experimental configuration of RXS measurement to investigate magnetic satellite peaks around the fundamental Bragg spots (0, 4, 0) and (0, 4, 4), which is the same as the one employed in Figs. 3a and b in the main text. Scattering plane is always perpendicular to the [100] axis. $\mathbf{k}_i$ and $\mathbf{k}_f$ are the propagation vectors of incident and scattered X-rays. In (a) and (b), $\mathbf{k}_i$ is almost parallel to the $[0\bar{1}1]$ and $[0\bar{1}0]$ directions, respectively. c,d) Line profiles for $(0, 4-\tau, 0)$ and $(0, 4-\tau, 4)$ scans corresponding to $\mathbf{Q}_2$ ($\perp$ $\mathbf{H}$), measured at 35 K with $\mu_0 H = 5.4$ T applied along the [100] direction (phase V). e) Schematic illustration of the fan magnetic structure.

magnetic reflections at $(0,4,4) \pm \mathbf{Q}_2$ with the setup shown in Supplementary Fig. S6b, where $\mathbf{k}_i$ is almost parallel to the $[0\bar{1}0]$ direction. In this case, $I_{\pi-\pi'}$ and $I_{\pi-\sigma'}$ mainly reflect the [100] and [010] component of $\mathbf{m}_\mathbf{Q}$, respectively. Supplementary Fig. S6d indicates the line scan profile of $I_{\pi-\pi'}$ and $I_{\pi-\sigma'}$ for $\mathbf{Q}_2$. The observed absence of $I_{\pi-\pi'}$ and $I_{\pi-\sigma'}$ intensity demonstrates that $\mathbf{m}_{\mathbf{Q}_2}$ contains neither [010] nor [100] component. Since the aforemen-

tioned results for $(0, 4, 0) \pm \mathbf{Q}_2$ (Supplementary Fig. S6c) suggest that the projection of $\mathbf{m}_{\mathbf{Q}_2}$ along the $[0\bar{1}1]$ direction is not zero, $\mathbf{m}_{\mathbf{Q}_2}$ should possess the [001] component. On the basis of the above results, we have concluded that the spin texture in the phase V can be approximately described by

$$\mathbf{m}(\mathbf{r}) \propto [(0, 0, m_c) \exp(i\mathbf{Q}_2 \cdot \mathbf{r}) + c.c.] + M_0(1, 0, 0), \tag{S1}$$

i.e., the superposition of sinusoidally modulated spin component along the [001] direction and the uniform ferromagnetic component along the $\mathbf{H} \parallel [100]$ direction (Supplementary Fig. S6e). This represents the fan magnetic structure, where the local magnetic moment along the [100] direction shows a slight periodic tilting toward the $\pm$[001] directions as indicated in Fig. 1h in the main text.

6. MAGNETIC STRUCTURES IN THE PHASES I AND III FOR H $\parallel$ [001]

Magnetic phase diagram of $GdRu_2Si_2$ for $\mathbf{H} \parallel [001]$ has previously been investigated by a part of the present authors in Ref. [S3]. In that work, the resonant X-ray scattering has been performed for the phases I, II and III, where the magnetic satellite reflections corresponding to $\mathbf{Q} = (q, 0, 0)$ and $\mathbf{Q} = (0, q, 0)$ are always observed. Such four-fold scattering patterns can represent either a double-Q state or multiple-domain of single-$\mathbf{Q}$ states with $\mathbf{Q} \parallel [100]$ and $\mathbf{Q} \parallel [010]$. In Ref. [S3], only the phase II has been identified as the double-$\mathbf{Q}$ state, and the phases I and III were tentatively assigned as single-$\mathbf{Q}$ states. Nevertheless, our present results in the main text demonstrate that the latter interpretation must be revised, and the phases I and III also represent the double-$\mathbf{Q}$ state.

To reconsider the previously obtained data, the results of polarization analysis for the RXS experiments in the phases I and III for $\mathbf{H} \parallel [001]$ are summarized in Supplementary Fig. S7. Here, the magnetic satellite reflections at $(2, 4, 0) \pm \mathbf{Q}$ were investigated with the setup shown in Supplementary Figs. S7a and S7b. In this configuration, the propagation vector of incident x-ray $\mathbf{k}_i$ is almost parallel to the [010] direction, and Eq. (2) in the main text suggests that $I_{\pi-\pi'}$ and $I_{\pi-\sigma'}$ mainly reflect the [001] and [010] components of $\mathbf{m}_{\mathbf{Q}}$, respectively.

Supplementary Figs. S7c and S7d indicate the line scan profiles in the phase I at 0 T measured for $\mathbf{Q} = (q, 0, 0)$ and $\mathbf{Q} = (0, q, 0)$, respectively. Here, we observe two magnetic

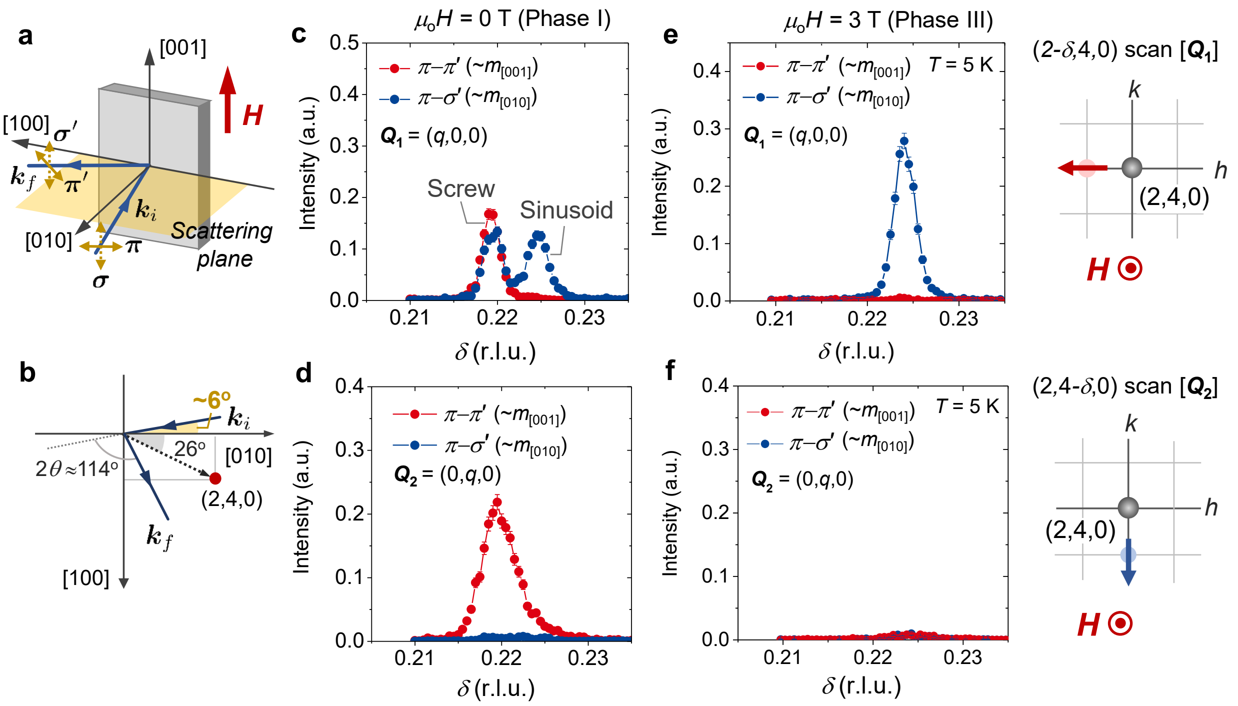

FIG. S7. Polarization analysis of RXS profile in the phase I and III under $\mathbf{H} \parallel [001]$. a,b) Experimental configuration of RXS measurement to investigate magnetic satellite peaks around the fundamental Bragg spots (2, 4, 0). Scattering plane is always perpendicular to the [001] axis. $\mathbf{k}_i$ and $\mathbf{k}_f$ are the propagation vectors of incident and scattered X-ray, respectively, and $\mathbf{k}_i$ is almost parallel to the [010] direction. c,d) Line profiles for $(2-\delta, 4, 0)$ and $(2, 4-\delta, 0)$ scans corresponding to $\mathbf{Q}_1$ and $\mathbf{Q}_2$, measured at 5 K with $\mu_0 H = 0$ T (i.e. phase I). e,f) The corresponding data measured with $\mu_0 H = 3$ T applied along the [001] axis (i.e. phase III). These data are reproduced from Ref. [S3].

satellite peaks at $q \sim 0.219$ and $q \sim 0.224$, in consistent with Fig. 2 in the main text. For the magnetic satellite peak at $q \sim 0.219$, the co-existence of $I_{\pi-\pi'}$ and $I_{\pi-\sigma'}$ in Supplementary Fig. S7c indicates the presence of [001] and [010] modulating spin components for $\mathbf{Q} \parallel [100]$. Likewise, the observed presence of $I_{\pi-\pi'}$ (absence of $I_{\pi-\sigma'}$) in Supplementary Fig. S7d indicates the presence of [001] (absence of [010]) modulating spin component for $\mathbf{Q} \parallel [010]$. These results suggest that the magnetic satellite reflection at $q \sim 0.219$ represents the screw spin modulation, where the neighboring magnetic moments rotate within a plane perpendicular to the magnetic modulation vector $\mathbf{Q}$. On the other hand, for the magnetic

satellite peak at $q \sim 0.224$, the absence of $I_{\pi-\pi'}$ (existence of $I_{\pi-\sigma'}$) in Supplementary Fig. S7c indicates the absence of [001] (presence of [010]) modulating spin component for $\mathbf{Q} \parallel [100]$. Likewise, the observed absence of $I_{\pi-\pi'}$ and $I_{\pi-\sigma'}$ in Supplementary Fig. S7d indicates that neither [001] nor [010] modulating spin component exists for $\mathbf{Q} \parallel [010]$. These results suggest that the satellite reflection at $q \sim 0.224$ represents the sinusoidal modulation of in-plane spin component normal to both the $\mathbf{Q}$-direction and the [001] axis. (Note that the latter magnetic reflection at $q \sim 0.224$ has originally been assigned as the contamination of different magnetic phase in Ref. [S3].) By considering the double-$\mathbf{Q}$ nature of phase I as demonstrated in the main text, the resultant spin texture can be approximately described as

$$\mathbf{m}(\mathbf{r}) \propto [(0, m_b, im_c)\exp(i\mathbf{Q}_1 \cdot \mathbf{r}) + (m_a, 0, 0)\exp(i\mathbf{Q}_2 \cdot \mathbf{r})] + c.c., \tag{S2}$$

i.e. the superposition of screw and sinusoidal spin modulations with $\mathbf{Q}_1 = (0.219, 0, 0)$ and $\mathbf{Q}_2 = (0, 0.224, 0)$, respectively (Fig. 1c in the main text). Since it represents an anisotropic double-$\mathbf{Q}$ state, two equivalent magnetic domains associated with four-fold symmetry breaking can coexist for $\mu_0 H = 0$ T.

The similar measurement has also been performed in phase III at 3 T for $\mathbf{H} \parallel [001]$ with the same experimental configuration, and the results are summarized in Supplementary Figs. S7e and S7f. The absence of $I_{\pi-\pi'}$ (existence of $I_{\pi-\sigma'}$) in Supplementary Fig. S7e indicates the absence of [001] (presence of [010]) modulating spin component for $\mathbf{Q} \parallel [100]$. On the other hand, the observed absence of $I_{\pi-\pi'}$ and $I_{\pi-\sigma'}$ in Supplementary Fig. S7f indicates that the neither [001] nor [010] modulating spin component exist for $\mathbf{Q} \parallel [010]$. These results suggest that $\mathbf{Q}$ represents the transverse sinusoidal modulation of in-plane spin component (normal to both the $\mathbf{Q}$-direction and the [001] axis). By considering the isotropic double-$\mathbf{Q}$ nature of the phase III (derived from the square lattice manner of charge density modulation detected by the previous STM measurements[S4]), the resultant spin texture can be approximately given by

$$\mathbf{m}(\mathbf{r}) \propto [(0, m_b, 0)\exp(i\mathbf{Q}_1 \cdot \mathbf{r}) + (m_a, 0, 0)\exp(i\mathbf{Q}_2 \cdot \mathbf{r}) + c.c.] + M_0(0, 0, 1), \tag{S3}$$

which represents the square vortex lattice described by the superposition of two sinusoidally modulated spin components (Fig. 1e in the main text).

* khanh.nguyen@riken.jp (N.D.K.); seki@ap.t.u-tokyo.ac.jp (S.S.)

[S1] F. R. Lux *et al.*, Phys. Rev. Lett. **124**, 096602 (2020).

[S2] A. Garnier *et al.*, J. Magn. Magn. Mater. **140**, 899 (1995).

[S3] N. D. Khanh *et al.*, Nature Nanotech. **15**, 444 (2020).

[S4] Y. Yasui *et al.*, Nature Commun. **11**, 5925 (2020).
